# Evaluating differences in the clinical impact of a free online weight loss programme, a resource-intensive commercial weight loss programme and an active control condition: a parallel randomised controlled trial

**DOI:** 10.1186/s12889-019-8061-x

**Published:** 2019-12-23

**Authors:** Aidan Q. Innes, Greig Thomson, Mary Cotter, James A. King, Niels B. J. Vollaard, Benjamin M. Kelly

**Affiliations:** 1 Nuffield Health Research Group, Nuffield Health, Ashley Avenue, Epsom, Surrey, KT18 5AL UK; 2 Faculty of Health, Psychology and Social Care, Manchester Metropolitan University, Manchester, M15 6GX UK; 3 National Centre for Sport and Exercise Medicine, Loughborough University, Loughborough, LE11 3TU UK; 4 National Institute for Health Research Leicester Biomedical Research Centre, Leicester, LE3 8QD UK; 5 Faculty of Health Sciences and Sport, University of Stirling, Stirling, FK9 4LA UK

**Keywords:** Weight loss, Exercise, Weight reduction programs

## Abstract

**Background:**

Finding effective intervention strategies to combat rising obesity levels could significantly reduce the burden that obesity and associated non-communicable diseases places on both individuals and the National Health Service.

**Methods:**

In this parallel randomised-controlled trial, 76 participants who are overweight or obese (50 female) were given free access to a fitness centre for the duration of the 12-week intervention and randomised to one of three interventions. The commercial intervention, the Healthy Weight Programme, (HWP, *n* = 25, 10/15 men/women) consisted of twelve 1-h nutrition coaching sessions with a nutritionist delivered as a mixture of group and 1 to 1 sessions. In addition, twice-weekly exercise sessions (24 in total) were delivered by personal trainers for 12 weeks. The **NHS** intervention (*n* = 25, 8/17 men/women) consisted of following an entirely self-managed 12-week online NHS resource. The **GYM** intervention (*n* = 26, 8/18 men/women) received no guidance or formal intervention. All participants were provided with a gym induction for safety and both the NHS and GYM participants were familiarised with ACSM physical activity guidelines by way of a hand-out.

**Results:**

The overall follow-up rate was 83%. Body mass was significantly reduced at post-intervention in all groups (HWP: *N* = 18, − 5.17 ± 4.22 kg, NHS: *N* = 21–4.19 ± 5.49 kg; GYM: *N* = 24–1.17 ± 3.00 kg; *p* < 0.001) with greater reductions observed in HWP and NHS groups compared to GYM (*p* < 0.05). Out with body mass and BMI, there were no additional statistically significant time x intervention interaction effects.

**Conclusions:**

This is the first study to evaluate the efficacy of both a free online NHS self-help weight-loss tool and a commercial weight loss programme that provides face-to-face nutritional support and supervised exercise. The findings suggest that both interventions are superior to an active control condition with regard to eliciting short-term weight-loss.

**Trial registration:**

ISRCTN Registry - ISRCTN31489026. Prospectively registered: 27/07/16.

## Background

Responsible for nearly two thirds of deaths worldwide [1], non-communicable diseases (NCD’s) represent a significant global risk. In the UK, the probability of dying between the ages of 30 and 70 years from one of the four main NCDs is estimated to be ~ 12% [2]. In England, it is estimated that at least 1 in 20 people has type-2-diabetes (T2DM) [3], with future trends predicting to rise by nearly a third to over four million diagnosed cases by 2025 [4]. In Scotland alone, where two thirds of adults are either overweight or obese, almost 20% of adults over the age of 16 years have some form of cardiovascular disease or T2DM [5].Obesity places a significant financial burden upon the National Health Services (NHS). Increased incidence of obesity parallels that of NCD’s, with excess weight contributing to approximately 44% of the global T2DM burden [6]. With co-morbidities such as hypertension [7] and dyslipidaemia [8], augmented by obesity, there is a pressing need for effective interventional strategies. Previous projections have suggested that reducing body mass index (BMI) by 1% across the UK population (equivalent to 1 kg weight loss) would substantially reduce burden, saving up to 202,000 incident cases of T2DM and 122,000 cases of cardiovascular diseases over 20 years [9].

Guidelines recommend that primary care physicians in Britain identify people with obesity and offer clinical management [10] but few options for treatment exist in traditional primary care settings. Data from NHS-led interventions is sparse. Research has suggested that the 12-week ‘Size down Programme’; an NHS group-based programme led by food advisers recruited from the local community, achieves significant weight loss, similar to competitor groups (Weight Watchers, Slimming World, Rosemary Conley, All *n* = 100) [11]. The NHS also provides a free, online, self-help tool [12]. This weight loss plan was developed in association with The British Dietetic Association, and according to the NHS website, has been downloaded more than 4 million times as of August 2019. The plan involves downloading and following 12 weekly information packs which provide advice on both healthy eating and physical activity covering topics including: portion control, building-up physical activity, food swaps, comfort eating and long-term maintenance. Although this calorie-restricted diet plan can be expected to be effective if adhered to, it remains uncertain whether an online programme will be sufficiently motivating to ensure adherence. It has been suggested that supervised diet plans have a greater chance of establishing and maintaining weight loss [13]. To our knowledge, the NHS weight loss plan has not previously been validated in any cohort, so questions remain about the effectiveness of this intervention.

To improve motivation and adherence, many commercial programmes share a similar structure of once-weekly group sessions ranging from 60 to 90 min. Educational content within commercial programmes is predominantly focussed on dietary manipulation and tracking, with energy deficit the central physiological component to weight loss. Generally, activity is encouraged and is usually guided via measurable metrics such as steps. Jebb and colleagues [14] demonstrated that following a 12-month commercial weight loss intervention participants had increased odds of losing 5 and 10% of initial body weight in comparison to participants receiving standard care (weight loss advice from a primary care professional following national guidelines of the country of the participant; UK, Germany or Australia). A large (*N* = 29,326) participant data set from NHS referrals to a commercial weight loss programme identified that 57% of participants lost at least 5% of initial body weight with 12% losing 10% of initial body weight after just 12 sessions [15], with a number of studies replicating comparable findings across similar if not identical commercial interventions [16–20]. The ‘Healthy Weight Program’ (HWP) is a commercial, individually tailored 12-week intervention that provides both exercise and nutritional support. Dedicated face-to-face exercise coaching is delivered by personal trainers and face-to-face nutrition coaching is delivered by registered nutritionists across multiple days of the week with the purpose of eliciting lifestyle and behaviour change to improve health and wellbeing in individuals. To date no research has compared a commercial intervention that combines intensive face-to-face support targeting both nutritional and exercise interventions to target weight loss with a less resource-intensive programmes such as the free online NHS weight loss tool.

The primary aim of the present study was to evaluate the effectiveness of reducing body mass through the scalable NHS resource compared to a commercial resource-intensive weight-loss intervention; as well as a ‘no-advice’ comparator group. The secondary aim of the present study was to evaluate the effectiveness of both interventions at improving health indicators related to obesity. We hypothesised that the HWP programme would result in significantly greater losses in body mass compared to the NHS weight loss programme. We further hypothesised that both programmes would elicit greater weight-loss than the ‘no-advice’ comparator group.

## Methods

### Participants

Participants for this parallel-randomized control trial were recruited from the local community near to the trial site (Glasgow, UK) via various advertising approaches including email, online features and social media in July and August 2016. People were directed to a purpose-built web page which provided greater detail, inclusion/exclusion criteria and an online contact form. Participant inclusion criteria included: aged between 18 and 50 years; a body mass index (BMI) between 30 and 45 kg/m^2^; not currently regularly exercising assessed as ‘low’ via categorical scoring of the International Physical Activity Questionnaire [21]; not currently dieting nor have done so recently; not a current member of any Nuffield Health Fitness and Wellbeing centres; able to commit to visiting the trial sites 3–4 times per week for the duration of the intervention; not pregnant or lactating; not undergoing treatment for, possession or diagnoses of any metabolic or cardiovascular disease and previous surgical procedures for weight loss. Participants with controlled hypertension remained eligible for the trial. Individuals with a blood pressure (BP) of > 140/90 mmHg were eligible to proceed upon approval and consent from their registered general practitioner. Pre-screening of participants took place in July and August 2016 with the interventions taking place from September 2016 until January 2017 depending upon participant start date.

Following initial screening, 76 participants (26 male, 50 female) were invited to proceed to the intervention stage and provided written informed consent. Participants were block randomised by computer programme (https://www.randomizer.org) to one of three interventions: Healthy Weight Programme**™** (HWP), National Health Service programme (NHS), or gym only comparator group (GYM). Due to the nature of the intervention, blinding was not possible. The University of Bath Research Ethics Advisory Committee approved this study (ref: EP 15/16259/283). Following completion of the intervention phase, participants in all three groups were given full access to a Nuffield Health Fitness & Wellbeing Centre for 12 months to assist them in achieving and maintaining any reduction in body mass and as a reimbursement for their time during the intervention. The CONSORT reporting guidelines were used [22].

#### Healthy weight Programme

The 12-week HWP intervention consisted of ten nutrition coaching sessions and 20 exercise sessions. The ten 1-h nutrition sessions were delivered by a registered nutritionist and consisted of a mix of one-to-one appointments and group classes. Two sessions were reserved for individual progress evaluations, specifically at weeks 6 and 10. Core themes included hunger and portions size, emotional eating, effects of sleep and stress, fuelling exercise, common weight loss myths, snacking, goal setting and meal planning. In addition, qualified exercise professionals provided twice-weekly exercise sessions for 10 weeks, starting at 30 min per session and increasing to 45 min by the end of the trial. Like the nutrition intervention, 2 weeks were reserved for individual progress evaluations. Sessions included indoor cycling, body weight circuit training, body pump classes and high-intensity interval training. Outside of structured fitness sessions, participants had free access to the swimming pool, gym and fitness classes but were not allowed to access personal training other than what was provided as part of the intervention.

#### NHS Programme

The 12-week NHS intervention is an entirely self-managed online resource [12]. Participants were encouraged to utilise the broader NHS choices network and associated online tools and apps such as recipe finder, meal planner, calorie tracker and a moderated weight loss social forum. In brief, this intervention involved participants downloading weekly modules from the NHS website within which standardised tasks and guidance were detailed. Content included information on fibre consumption, portion control, exercise preparation, building-up physical activity, motivation strategy, breakfast advice, workplace wellbeing, cravings, alcohol awareness, plateaus, peer pressure, dining out, foreign foods, breaking down perceived barriers to change, food swaps, comfort eating and long-term maintenance. Participants received a 2-weekly call from the research team to resolve any technical/access issues. At no stage was additional coaching or feedback provided. For reasons of health and safety, participants were provided with an induction to the gym, and given full access to the gym and swimming pool for the duration of the intervention but were not allowed to access personal training during this time. Participants were familiarised with the basic American College of Sports Medicine (ACSM) physical activity guidelines [23] and how this would translate to the fitness and wellbeing centre by way of a handout (Appendix 2).

#### Gym only

The gym only group received no guidance or formal intervention. Following baseline assessments, participants were provided with an induction to the gym which acted as a health and safety measure. Participants were familiarised with the basic ACSM physical activity guidelines [23] and how this would translate to the fitness and wellbeing centre by way of a handout (Appendix 2) but were given no additional advice thereafter.

### Data collection

All data were collected at baseline and 12 weeks and were taken following a 12 h overnight fast. Blood analyses were done using venous blood samples collected via venepuncture of the antecubital vein. Blood samples were collected into vacutainers™ (Becton Dickinson, Plymouth, UK; SST™ II / 2KEDTA) for analysis of plasma insulin, blood lipid profile, fasting blood glucose and HbA1C. All samples were temporarily stored at 4 °C and analysed within 24 h of collection. Plasma total cholesterol and triglycerides (free glycerol blank subtracted) were measured enzymatically using established clinical chemistry laboratory methods [24, 25] (Nuffield Health, Glasgow, UK). High-density lipoprotein cholesterol (HDL-C) was measured by liquid selective detergent followed by enzymatic determination of cholesterol [26]. Low-density lipoprotein cholesterol (LDL-C) was calculated according to Friedewald et al. [27]. Total plasma insulin in serum was measured by radioimmunoassay [28] and blood glucose was measured using a modification of the glucose oxidase/peroxidase method [29, 30]. A HPLC-ESI/MS approach was utilized to measure blood HbA1c concentrations [31]. An estimation of insulin resistance and β-cell function was provided via the homeostasis model assessment as described elsewhere (HOMA-IR [32],).

Anthropometric measurements were made according to the recommendations of the International Standards for Anthropometric Assessment (ISAK) [33]. Fat mass and fat-free mass were assessed by bioelectrical impedance analysis in accordance with manufacturer recommendations (Bodystat 1500, Bodystat Ltd., UK). Blood pressure and resting heart rate were measured via automated blood pressure cuff (Omron M3 Comfort, Omron Corporation, Japan) in accordance with the European Society of Hypertension guidance [34]. Mean arterial pressure (MAP) was recorded and defined as [(2 x diastolic) + systolic]÷3. Assessment of cardiovascular disease risk and T2DM risk assessed using the QRisk2 risk calculator [35] and QDiabetes [36] risk calculator respectively.

### Statistical analysis

Data are presented as mean ± SD. Statistical analyses were performed using IBM SPSS Statistics 23. As the aim of the study was to establish differences in the effects of two interventions vs. a control condition rather than establishing the effect of treatment assignments per se, we chose to employ Per Protocol analysis rather than Intention to Treat analysis. The primary outcome measure was the change in body mass from baseline to follow-up. To detect differences between the effects of the interventions on body mass with a medium effect size of f = 0.25 we required 18 participants in each group to achieve a power of 95% and α = 0.05. To allow for drop out of participants during the study period we aimed to recruit a sample size of 25 participants in each group. A two-way mixed ANOVA (intervention x time) was performed to determine the effects of the interventions on the outcome measures, with the intervention x time interaction effect as the main statistic of interest. In the case of significant main effects, *post-hoc* comparisons were performed using Fishers LSD (i.e. uncorrected paired t-tests) since there is no inflation of type 1 error rates following a significant main effect when only three comparisons are being made [37]. Alpha was set at 0.05.

## Results

Of the 76 participants who started (*n* = 25, 25, 26 for HWP, NHS and GYM respectively), 13 withdrew citing a declination to continue in the study (*n* = 7, 4, and 2 for HWP, NHS and GYM respectively) (see Appendix 1 for participant flow diagram). Table 1 presents the characteristics of all starting participants and participants who completed the study and were included in the Per Protocol analysis. There were no significant differences between groups at baseline.

**Table 1:** Participant Characteristics

	HWP		NHS		GYM	
	Starting Cohort *N* = 25	Per Protocol Analysis *N* = 18	Starting Cohort *N* = 25	Per Protocol Analysis *N* = 21	Starting Cohort *N* = 26	Per Protocol Analysis *N* = 24
Men / women	10 / 15	8 / 10	8 / 17	7 / 14	8 / 18	7 / 17
Age (y)	40 ± 8 (20–50)	43 ± 5 (33–50)	37 ± 8 (23–50)	37 ± 8 (23–50)	38 ± 7 (20–47)	37 ± 8 (20–47)
Height (m)	1.72 ± 0.10 (1.57–1.91)	1.72 ± 0.10 (1.57–1.86)	1.70 ± 0.11 (1.52–1.87)	1.71 ± 0.11 (1.52–1.90)	1.68 ± 0.09 (1.54–1.87)	1.68 ± 0.09 (1.54–1.87)
Body Mass (kg)	106.06 ± 15.66 (82.50–141.00)	106.38 ± 14.90 (89.50–140.00)	102.53 ± 16.79 (77.00–145.10)	103.13 ± 16.92 (77.00–145.10)	98.52 ± 13.32 (80.20–126.80)	98.98 ± 13.54 (80.20–126.80)
BMI (kg/m^2^)	36.01 ± 3.26 (30.00–41.20)	35.86 ± 3.42 (29.67–40.47)	35.31 ± 3.40 (29.71–41.76)	35.25 ± 3.51 (29.71–40.19)	34.78 ± 2.90 (30.07–40.11)	34.85 ± 2.97 (30.07–40.11)

Values shown are means ± SD (range)

**Fig. 1:**
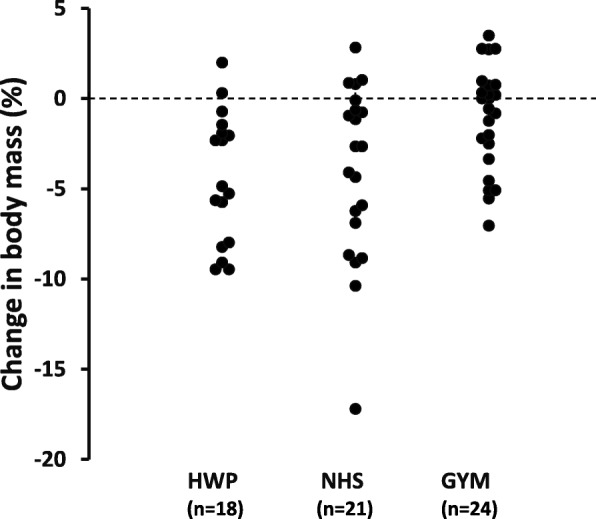
Individuals’ post intervention changes in body mass between groups

Main effects of time were observed (Table 2), with reductions in body mass (*p* < 0.001), BMI (*p* < 0.05), waist (*p* < 0.001) and hip circumference (*p* < 0.001), absolute body fat (kg, *p* < 0.001), fat free mass (*p* < 0.01), plasma triglycerides (*p* < 0.01), LDL-C (*p* < 0.01), total cholesterol:HDL cholesterol ratio (*p* < 0.05), fasting blood glucose (*p* < 0.05), HbA1C (*p* < 0.05), and 10-year cardiovascular disease risk according to the QRISK-2 score (*p* < 0.01), and an increase in plasma HDL-C (*p* < 0.01). No changes from pre- to post-intervention were observed for percentage body fat, total cholesterol, insulin, HOMA-IR, and T2DM risk (Q-Diabetes). A significant intervention x time interaction effect was observed for both body mass (*p* < 0.01) and BMI (*p* < 0.05). Greater reductions in body mass were observed in HWP (5%, *p* < 0.001) and NHS (4%, *p* < 0.001) compared to GYM (1%) with no difference between the HWP and NHS interventions (Fig. 1).

**Table 2:** Baseline and follow-up changes in physiological markers and disease risk predictions following 12-week intervention in participants who completed both the baseline and follow-up testing only

		HWP		NHS		GYM		Δ Change	
		Baseline ± SD	Follow-up ± SD	Baseline ± SD	Follow-up ± SD	Baseline ± SD	Follow-up ± SD	Time	Time x Intervention
Anthropometrics	Body Mass (kg)	**106.38 ± 14.90**	**101.21 ± 14.16**	**103.13 ± 16.92**	**98.94 ± 16.97**	**98.98 ± 13.54**	**97.81 ± 12.61**	**< 0.001**	< 0.01
	Body Mass Index (kg.m^2^)	**35.86 ± 3.41**	**34.16 ± 3.73**	**35.25 ± 3.51**	**33.82 ± 3.86**	**34.85 ± 2.97**	**34.50 ± 3.20**	**< 0.01**	< 0.05
	Waist Circumference (cm)	**108.6 ± 12.3**	**101.9 ± 8.8**	**107.2 ± 12.0**	**96.4 ± 12.2**	**104.9 ± 10.2**	**98.2 ± 11.3**	**< 0.001**	NS
	Hip Circumference (cm)	**119.5 ± 10.2**	**114.6 ± 9.9**	**119.0 ± 10.3**	**113.7 ± 11.0**	**117.8 ± 6.2**	**115.0 ± 7.5**	**< 0.001**	NS
	Body Fat (%)	40.9 ± 9.1	39.4 ± 8.9	39.4 ± 8.9	39.2 ± 7.5	40.3 ± 7.7	40.2 ± 7.9	NS	NS
	Body Fat (kg)	43.1 ± 9.3	39.4 ± 10.0	40.5 ± 11.1	38.7 ± 9.9	39.8 ± 9.0	39.2 ± 9.3	NS	NS
	Fat Free Mass (kg)	**63.3 ± 15.1**	**60.5 ± 12.8**	**62.1 ± 14.6**	**60.4 ± 13.5**	**59.4 ± 12.3**	**58.2 ± 10.9**	**< 0.01**	NS
Hemodynamics	Systolic Blood Pressure (mmHg)	138 ± 19	134 ± 14	129 ± 12	129 ± 11	129 ± 11	130 ± 8	NS	NS
	Diastolic Blood Pressure (mmHg)	91 ± 12	87 ± 8	82 ± 7	82 ± 8	81 ± 7	82 ± 7	NS	NS
	Mean Arterial Pressure (mmHg)	106 ± 14	104 ± 10	98 ± 8	99 ± 9	97 ± 7	97 ± 11	NS	NS
	Resting Heart Rate (beat/min)	70 ± 10	70 ± 9	70 ± 11	68 ± 9	68 ± 8	65 ± 9	NS	NS
Hematology	Fasting Blood Glucose (mmol/L)	**5.27 ± 0.66**	**5.20 ± 1.09**	**5.20 ± 0.51**	**4.76 ± 0.59**	**5.08 ± 0.50**	**4.90 ± 0.39**	**< 0.05**	NS
	Insulin (mlU/L)	16 ± 24	10 ± 6	10 ± 6	8 ± 4	20 ± 47	9 ± 5	NS	NS
	HOMA-Insulin Resistance (au)	3.8 ± 5.2	2.3 ± 1.2	2.4 ± 1.6	1.8 ± 1.0	5.0 ± 11.6	4.7 ± 11.6	NS	NS
	Triglycerides (mmol/L)	2.14 ± 1.49	1.68 ± 0.99	1.84 ± 1.22	1.4 ± 0.8	1.47 ± 0.87	1.40 ± 1.05	0.01	NS
	Total Cholesterol (mmol/L)	5.29 ± 0.84	4.89 ± 1.03	5.43 ± 0.80	5.13 ± 0.74	5.00 ± 1.07	5.07 ± 1.46	NS	NS
	HDL-Cholesterol (mmol/L)	**1.28 ± 0.30**	**1.31 ± 0.28**	**1.39 ± 0.40**	**1.49 ± 0.42**	**1.31 ± 0.36**	**1.38 ± 0.33**	**< 0.001**	NS
	Total: HDL Cholesterol Ratio	**4.4 ± 1.5**	**3.9 ± 1.2**	**4.3 ± 1.6**	**3.8 ± 1.3**	**4.0 ± 1.3**	**4.0 ± 2.1**	**< 0.05**	NS
	LDL-Cholesterol (mmol/L)	**3.31 ± 1.03**	**2.92 ± 0.95**	**3.26 ± 0.68**	**3.07 ± 0.77**	**3.08 ± 1.12**	**3.00 ± 1.02**	**< 0.01**	NS
	HbA1C DCCT (%)	**5.4 ± 0.7**	**5.2 ± 0.4**	**5.2 ± 0.4**	**5.1 ± 0.2**	**5.1 ± 0.3**	**5.1 ± 0.3**	**< 0.05**	NS
	Hba1C IFCC (mmol/mol)	**36 ± 7**	**33 ± 4**	**33 ± 4**	**32 ± 2**	**32 ± 4**	**33 ± 4**	**< 0.05**	NS
Disease Risk	QRISK2 Prediction (%)	**2.8 ± 2.0**	**2.3 ± 1.7**	**1.5 ± 1.5**	**1.3 ± 1.4**	**1.3 ± 1.1**	**1.3 ± 1.1**	**0.001**	NS
	QDiabetes Risk Prediction (%)	8.4 ± 5.3	7.2 ± 4.7	5.6 ± 5.1	6.6 ± 11.2	5.0 ± 4.1	4.6 ± 3.9	NS	NS

**Bold** denotes significant main effect of time. **Δ Change (*p*-Value)** refers to difference between pre and post intervention for all groups (time). *N* = 18, 21 and 24 for HWP, NHS and GYM respectively. NS denotes Non-significant. Significance accepted as *p* < 0.05

## Discussion

The primary aim of this study was to evaluate the effectiveness of a resource-intensive commercial weight-loss programme versus a free online NHS weight-loss intervention and an active control condition. We hypothesised that the more resource-intensive commercial HWP intervention would result in superior reductions in body mass compared to the NHS intervention and GYM control. However, despite the low cost and ease of delivery, the free, online NHS intervention was comparable to the commercially available, resource-intensive HWP intervention in reducing body mass, with significantly greater losses in body mass observed in both interventions compared to the active-control GYM condition.

This is the first study to demonstrate the effectiveness of the free online NHS weight loss programme at enabling individuals to achieve reductions in body mass. The NHS resource has several useful additions on the associated web-page, including: automated support email sign-up, access to a weight-loss forum, a calorie-checker, a mobile app, and articles on success stories and tips to overcoming barriers. This volume of additional support options and the ability to access supportive content at the participants’ leisure may explain the similarities in results of the NHS intervention compared to the HWP intervention, which does not provide access to such content outside of the face-to-face sessions, although usage of additional content was not assessed in the current study. A large volume of content including exercise videos is available at the Nuffield Health website, but participants were not specifically made aware of this.

The UK Department of Health’s best practice guidance for weight management [38] advises that weight loss programmes must achieve measurable health outcomes. Specifically, weight loss programmes should ensure that interventions lead to an average weight loss of at least 3%, with at least 30% of participants losing at least 5% of their initial weight. This target was met in the HWP and NHS group, which further demonstrates the effectiveness of the NHS weight loss plan. Whilst both HWP and NHS groups achieved 5% weight loss in 50 and 38% of participants respectively, the GYM group marginally missed this target, achieving 3% weight loss in 29% of participants, which may suggest that the observed effects in the NHS and HWP groups was not merely because of offering free gym access.

Heshka and colleagues [17] previously demonstrated that weight loss was significantly greater following a commercial weight loss programme (Weight Watchers; − 4 kg) compared to a self-help programme (−1.5 kg). This is at odds with the current findings, with the commercial HWP group achieving 5.2 ± 4.2 kg weight loss (5%) and the NHS self-help group achieving 4.2 ± 5.5 kg weight loss (4%) with no statistical difference between the two groups. A study by Baetge et al. [39] compared a programme that incorporates both exercise and dietary interventions (Curves) versus programmes that provide a dietary intervention only but advocates for exercise (Weight Watchers, Jenny Craig and Nutrisystem) and a non-intervention control group, evaluated over a 12-week period in a population of participants who were overweight or obese. Data demonstrated significant reductions in body mass versus a no-intervention control for all groups. Briefly, these were translated in to the following percentage changes: Curves (a combined meal-plan and exercise intervention) -4.7%, Weight Watchers (a group based, points-orientated nutritional intervention) -4.8%, Jenny Craig at Home (an online nutrition-focussed intervention) -5.9%, and Nutrisystem Advance Select (a meal-plan based system with online support) -5.3%. The results of this study align to those of the present study where a combined exercise and nutrition intervention (HWP) was not superior to a nutrition intervention which advocates for exercise (NHS). Furthermore, a systematic review and meta-analysis of 26 studies examining remotely delivered standalone interventions to elicit eating behaviour change by Teasdale et al [40] found a small but significant positive effect on eating behaviour change compared to control groups. The authors conclude that, albeit small, standalone self-management or targeted feedback interventions – such as the online NHS programme - could have an appreciable impact at a population level.

A time x intervention interaction only existed for body mass and BMI; however, a main effect of time showed positive changes in several additional indices including: waist circumference, HDL cholesterol, LDL cholesterol, plasma triglycerides, and blood glucose. These findings suggest that all three conditions elicited an improvement in several health markers however the two main interventions were not superior to the active-control condition for any health marker out with body mass and BMI. Results in the present study are at odds with Baetge and colleagues [39] who showed significant pre to post improvements in the aforementioned following 12-weeks of weight-loss interventions compared to their control group. The present study and that of Baetge et al. [39] differ somewhat however in that the present study includes both males and females and an active control condition allowing for the assessment of both weight-loss interventions in a ‘real-world’ setting whereas Baetge and colleagues recruited women only and had a no-intervention control. In the case of the present study, although the primary aim was achieved, it is possible to postulate that the inclusion of the “real-world” control condition masked further between-group differences seen in other studies that used no-intervention control groups.

Current guidance from the National Institute Health and Care Excellence [41] recommends that individuals who are overweight or obese be referred to group rather than individual programmes due to cost effectiveness. This can include lifestyle weight management programmes delivered by the public, private or voluntary sector. The NHS intervention in the current study offers a potential opportunity that is scalable, cost effective and can acutely achieve clinically significant weight loss similar to commercial lifestyle weight management programmes that patients are referred to such as Weight Watchers [15] and slimming world.

### Strengths and limitations

The addition of an active-control group was a strength of the present study. We recruited a very motivated cohort who were randomised to three groups with varying levels of support. Providing the control group with free access to fitness facilities but no further support allowed for the evaluation of both interventions in a ‘real-world’ setting.

The duration of the present study (12 weeks) is acute and therefore longer-term outcomes are not known. A 6 and 12 –month follow-up was planned but was unable to be conducted due to operational constraints within the delivery organisation. This was unfortunate as a key question about the long-term maintenance of weight-loss following both interventions remains unanswered. Future work requires detailed objective monitoring of physical activity. Participants in the present study were given access to fitness facilities however an unforeseen error meant that usage data was not collected. The NHS resource has several additions on the associated web-page; although participants were made aware of this information, we were unable to track usage. It should also be noted that the results in the present study may be explained by the fact that the present study had a very motivated cohort given that participants volunteered to take part and therefore, results in the general population may differ.

The present study was too small to derive reliable estimates of adherence, and as such we chose to initially examine the effectiveness of the two interventions at inducing reductions in body mass. Now that we have established that the free, online NHS programme does not result in significantly lower reductions in body mass compared to a resource-intensive commercial programme, there is a need for future studies to establish possible differences in adherence and to utilise Intention to Treat analysis rather than Per Protocol analysis as used in the present study [42].

## Conclusion

This is the first study to evaluate the free online NHS self-help weight-loss tool and compare it to a commercial weight loss programme. Our findings suggest that the NHS weight loss tool is an effective intervention for reducing body mass in the short term, and that providing a more resource-intensive intervention does not necessarily yield additional benefits, at least in the short term. Both interventions were superior compared to the control group at eliciting a reduction in body mass. Although current guidelines regarding weight-loss recommend people are referred to group programmes due to their cost-effectiveness, the current study demonstrates clinically significant weight-loss can be achieved by a free, online, scalable, self-help website. Thus, clinicians and the NHS may have an alternative and effective option to support weight-loss, with potential additive benefits expected should a structured exercise environment be made available.

## Figures and Tables

**Fig. 2:**
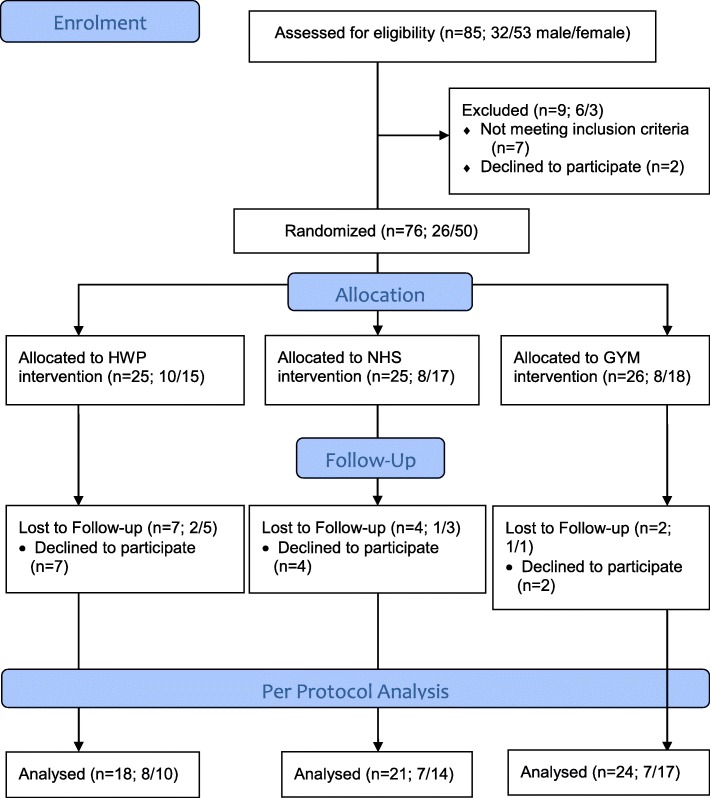
CONSORT flow diagram

## Data Availability

The data generated and/or analysed during the current study are available from the corresponding author on reasonable request.

## Appendix 2

### Gym-based Exercise Information Guide

#### Healthy Weight Study \ Nuffield Health Research Group

The American College of sports Medicine (ACSM) recommend that each person performs: 150 min of moderate of 75 min of vigorous intensity aerobic exercise across 7 days, as well as 2 sessions of whole body resistance exercises and at least 2 sessions working on flexibility.

#### 150 min of moderate intensity or 75 min of vigorous intensity aerobic exercise per week

- Aerobic exercise improves your hearts efficiency as a pump and when performed consistently, can reduce your risk of virtually all lifestyle related diseases.
  - ○ Moderate intensity – a 4–7 out of 10 in terms of perceived effort. You are likely to have increased breathing rate, perhaps be lightly sweating; however, you should be able to keep this intensity up for around an hour.
  - ○ Vigorous intensity – an 8 out of 10 at least in terms of intensity. You shouldn’t feel comfortable at this intensity. You’re likely to be sweating heavily and short of breath; however, you should be able to speak in short phrases.
    - ■ Example Moderate Intensity Exercises
      - Continuous swimming
      - Brisk Walking/light jogging
      - Continuous cycling/stepper/rowing/cross trainer
    - ■ Example Vigorous Intensity Exercises
      - Body Pump class
      - Metafit Class
      - Cycle Class
      - Interval training on cardio machines
      - Circuits Class
      - Boxing Fit Class

#### Whole body resistance training at least twice per week:

- Resistance training typically involves performing exercises against a force or weight for several repetitions per set. There are many variations of repetitions and sets but the general principles you should follow are:
  - ○ Perform at least 1 exercise per large muscle group such as:
    - ■ Leg Press Targets most muscles in your legs
    - ■ Chest Press Targets most muscles in your chest as well as arms
    - ■ Seated row Targets muscles in your upper and middle back and arms
  - ○ You can utilise fixed weight machines or free-weights depending on your experience. If you are inexperienced, fixed-weight machines will guide you safely.
  - ○ Keep track of the exercises you do, the weights, the repetitions and the sets you perform with a view to increased one of these aspects each week to continuously improve.
  - ○ Performing 3 sets of 12–15 repetitions will be sufficient to start with but you can increase/decrease the sets/reps as you feel the need to, but keep a note so you can improve next time you’re in the gym
  - ○ Some fitness classes will also count as resistance training such as body pump and circuits.

#### Perform at least two sessions of flexibility exercises per week:

- Working to improve and then maintain flexibility across all major muscle groups at least twice per week will help improve and maintain range of motion in your joints and may help reduce certain risks such as low back pain.
  - ○ Performing a yoga class is a great way to learn various stretches which you can then implement into your workout routine
  - ○ You can attend yoga classes for free during the study.

**Additional Information:** For the duration of the study, you will be unable to purchase Personal Training. For any questions or issues, please contact one of the researchers.

## Abbreviations

ACSM: American College of Sports Medicine
BMI: Body mass index
BP: Blood pressure
GYM: Gym-only comparator group
HDL-C: High-density lipoprotein cholesterol
HOMA-IR: Homeostatic modelling assessment for insulin resistance
HWP: Healthy Weight Programme
LDL-C: Low-density lipoprotein cholesterol
MAP: Mean arterial pressure
NCD’s: Non-communicable Diseases
NHS: National Health Service
T2DM: Type-2-diabetes
